# Dissolution from Ethylene Vinyl Acetate Copolymer Long-Acting Implants: Effect of Model Active Ingredient Size and Shape

**DOI:** 10.3390/pharmaceutics14061139

**Published:** 2022-05-27

**Authors:** Anne M. Gohn, Amy Nolte, Ethan Ravotti, Seth P. Forster, Morgan Giles, Nathan Rudd, Gamini Mendis

**Affiliations:** 1 School of Engineering, Penn State Behrend, 4701 College Drive, Erie, PA 16563, USA; aln5286@psu.edu (A.N.); err5206@psu.edu (E.R.); gmendis@psu.edu (G.M.); 2 Merck & Co., Inc., Rahway, NJ 07033, USA; seth_forster@merck.com (S.P.F.); morgan.giles@merck.com (M.G.); nathan.rudd@merck.com (N.R.)

**Keywords:** ethylene vinyl acetate, EVA, dissolution, migration, calcium carbonate, drug delivery, pharmaceutical, drug release

## Abstract

In recent pharmaceutical applications, an active pharmaceutical ingredient (API) can be mixed with a polymer material to yield a composite long-acting drug-delivery device. These devices boast higher patient compliance, localized drug delivery, and lower dosage concentrations, which can increase patient safety. As a laboratory-safe option, calcium carbonate (CaCO_3_) was used as a drug surrogate to mimic the release kinetics of a low-solubility API. The release of CaCO_3_ from a poly(ethylene vinyl acetate) (EVA) polymer matrix was studied in ultra-high-purity water. The geometry of CaCO_3,_ along with the manufacturing technique, was manipulated to study the implications on surrogate drug release. It was found that injection molding proved to yield higher burst release, due to higher pressures achievable during manufacturing. The extrusion process can affect the surface concentration of the pharmaceutical ingredient when extruded through a water bath, resulting in a lower initial burst concentration. Regarding CaCO_3_ geometry, the particle size was more critical than the surface area in terms of CaCO_3_ release. Larger particles showed a higher release rate, though they also displayed higher variability in release. These data can be used to engineer specific release profiles when designing composite formulations and manufacturing methods for pharmaceutical-drug-delivery applications.

## 1. Introduction

Plastics often have additives, fillers, and reinforcing agents added to their formulation to alter the mechanical, visual, stability, and thermal properties of the bulk material [1]. In some cases, the leaching of the additives or fillers from the polymer matrix is an undesirable trait, for example, in food contact [2] or environmental exposure scenarios [3,4,5]. However, sometimes the dissolution of the additives can be engineered for specific purposes, for example pharmaceutical-drug-delivery devices [6,7,8,9] or membranes for water purification [10]. Here, we aim to focus on engineering specific release profiles for implantable drug-delivery devices for the pharmaceutical industry.

Dissolution and diffusion of the additives in the polymer matrix are distinct mechanisms; dissolution is related to solubility, where the additive must have some ability to dissolve in the fluid medium, and diffusion is where the additive presents a physical movement through a void network. In the work presented here, the authors chose calcium carbonate (CaCO_3_) as the filler medium for study, as it has a low solubility in water [11], and thus, can be used as a surrogate material to safely study low-solubility compounds used in pharmaceutical-drug-delivery devices. In long-acting implantable drug-release devices, it was found that biodegradable polymers including poly(lactic acid) (PLA) and poly(caprolactone) (PCL) showed faster active pharmaceutical ingredient (API) release than in non-biodegradable polymers such as poly(ethylene vinyl acetate) (EVA) [6]. Specifically, EVA is the material used as the polymer matrix for commercial long-acting drug-release implants, such as Implanon, Dapivirine–maraviroc, and NuvaRing [12]. In this copolymer, the drug release is a function of the vinyl acetate content [13], which determines the maximum crystallinity of the polymer phase [14,15]. The molecular weight of the polymer matrix can also affect the diffusion of the API, as it has been found that the increased molecular weight also increases chain entanglement, reducing the chain mobility. This leads to a reduction in free volume which permits molecule transfer through the polymer matrix [16].

Regardless of the polymer used as the matrix material, the API release kinetics show an initial burst release, where a high concentration of the API is immediately released into the surrounding solution, then the system slows to a pseudo-steady-state release profile [6]. The migration of the pharmaceutical ingredient is dependent on the development of a porous void network within the polymer that allows for connectivity between the additive particles [6]. Environmental factors such as pH can also affect the release rate, as acidity levels can influence the swelling of the polymer drug-delivery device [17]. Prior research regarding the manufacturing conditions producing extruded drug-delivery devices suggest that higher shear and stress increase the drug release rate [18,19]. Within the extrusion process, the melt temperature also proves to be a critical processing parameter, as a processing temperature set too low increases the occurrence of voids in the polymer matrix, and a processing temperature set too high increases the agglomeration of the API phase [20].

The authors hope that this research will help a variety of industries to engineer specific formulations for intentional additive release over time. This study focuses on how the size and shape of the additive material can affect dissolution and how it alters the base polymer physical and mechanical properties.

## 2. Materials and Methods

### 2.1. Materials

Ateva^®^ ethylene vinyl acetate (EVA) 1070 with 9 wt-% vinyl acetate content and a melt flow index of 2.8 g/10 min was obtained from Celanese to be used as the base polymer. To mimic the low solubility of specific pharmaceutical long-term drug-release implants, composites were made using CaCO_3_ of differing geometry. Several grades of CaCO_3_ were obtained from Specialty Minerals Inc. (New York, NY, USA) with a variety of particle sizes and shapes, as summarized in Table 1.

To create the composites, the EVA pellets and CaCO_3_ powder were dry-mixed at a 50:50 weight ratio. Melt compounding was performed on a Haake Rheodrive 4 twin screw extruder with 16 mm segmented screws and 25:1 L/D. A melt temperature of 180 °C and a screw speed of 50 RPM were employed for extrusion, and the extrudate was pulled through a water bath to cool and fed into a pelletizer to create pellets for molding.

**Figure 1:**
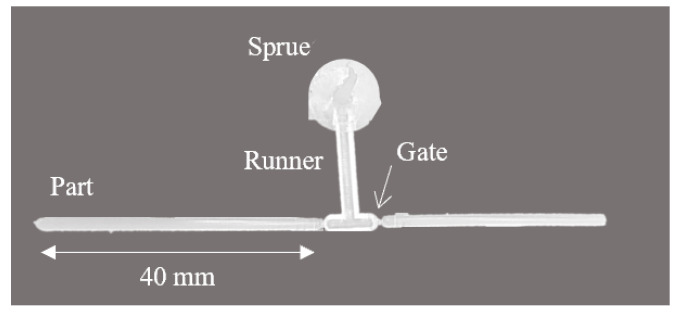
Injection-molded part geometry. A 2-cavity mold was used to fabricate rods 2 mm in diameter and 40 mm in length.

Samples of 2 mm diameter rods were then fabricated via injection molding and single-screw extrusion. Injection molding was performed on a BOY XS molding machine equipped with a 12 mm screw diameter, 19.7 L/D ratio, and 50 mm diameter barrel. The part geometry can be found in Figure 1, where a 2-cavity mold was used to fabricate rods. For the injection molding process, a barrel and hot sprue temperature of 180 °C, shot size of 14.5 mm, and injection velocity of 20 mm/s were used. The part geometry was filled to 95% of the maximum volume by shot size alone, then a holding pressure of 850 psi was applied for 3 s to compensate for shrinkage during melt solidification [21]. To compare dissolution characteristics as a function of the manufacturing conditions, samples were also fabricated via single-screw extrusion. A Brabender Plasticorder Rheometer with a 19 mm screw, 30:1 L/D ratio, and 3:1 compression ratio was used to fabricate the samples. A melt temperature of 180 °C, screw speed of 50 RPM, and die diameter of 3 mm were used. A puller was then used to draw-down the extrudate to a 2 mm diameter. Both the injection-molded and extruded specimen diameters were kept at a 2 mm ± 0.05 mm tolerance.

### 2.2. Instrumentation

Particle size analysis. A volume-based particle size analysis of the as-received CaCO_3_ powder was performed using a Mastersizer 3000 with Hydro MV wet-dispersion attachment from Malvern Instruments with the powders suspended in isopropyl alcohol (IPA). A small amount of dry powder was put directly into the Hydro MV unit with a constant stirrer speed of 1380 rpm in small increments until an obscuration rate of ~5% was achieved. The particle size distributions were calculated using the Mastersizer 3000 software (version V3.62) (Malvern Panalytical Ltd., Malvern, UK) using Mie theory. The refractive index and absorption of particles were set as 1.656 and 0.010, respectively (source: Malvern database for calcite). Five repetitions for each sample were analyzed, and the results reported are the average of all scans.

Scanning electron microscopy (SEM). Microscopy of the as-received CaCO_3_ powder and the cross sections of the fabricated samples were imaged using a FEI Quanta 650. Imaging was performed using a spot size, accelerating voltage, and chamber pressure of 3, 5 kV, and 20 Pa for the CaCO_3_ powder and 3, 10 kV, and 60 Pa for the polished cross sections, respectively. Sample cross sections were prepared with flow and perpendicular to flow by mounting in a cold-cast 2-part epoxy and polished using an Allied Met Prep 3 grinder/polisher. Samples were first polished using SiC grit starting with 320, 400, 600, 800, and 1200 with water, and then finely polished using an Allied 3 µm water-based poly diamond slurry on a Gold Label Pad, a 1 µm water-based poly diamond slurry on a White Label Pad, and the final polish was with an Allied 0.04 colloidal silica on a Final A pad.

Thermogravimetric Analysis (TGA). A Mettler Toledo TGA2 was used to evaluate the resulting loading level of CaCO_3_ in the prepared samples based on phase degradation. Samples of approximately 10 mg were heated at a rate of 10 °C/min to 900 °C under a nitrogen purge. A Huber minichiller 300 was used for thermal stabilization. Three samples of each composite were analyzed to obtain confidence in part-to-part variation. The results are reported with normalization to total sample weight.

Differential scanning calorimetry (DSC). A Mettler Toledo DSC2 equipped with a Thermo Scientific EK90/MT chiller was used to study the crystallinity content in the manufactured samples. The thermal profile used employed a heating rate of 10 °C/min from 25 °C to 120 °C, where the sample was held in the melt for 3 min to allow for thermal equilibrium. The sample was then cooled to 25 °C at 10 °C/min, where it was then held for 3 min. This protocol was repeated a second time. A nitrogen purge of 30 mL/min was used for all measurements to prevent sample oxidation, and samples of approximately 5 mg mass were used. The melting peak was integrated, and the percent crystallinity was calculated based on a heat of fusion of 293 J/g for a 100% crystalline sample [14].

Dissolution. The migration of CaCO_3_ from the EVA matrix over time was studied using an Labnet 211DHS shaking incubator. Samples were manually cut to 10 mm length from the injection-molded and extruded implants. If the samples were injection-molded, then the last 10 mm of the part were used at the end-of-fill location. Samples were placed in a 15 mL centrifuge tube in 10 mL of ultra-high-purity (UHP) water (18.2 MΩ) and kept fully submerged by an aluminum anchor weight designed to have minimal surface area impact. UHP water was obtained using an ELGA PURELAB flex 4. The incubator chamber was set to 37 °C to mimic human body temperature, and movement was programmed to a shaker speed of 30 and rotisserie power of 4. A micropipette was used to collect the solution at log-scale time intervals, and the solution was analyzed by inductively coupled plasma mass spectrometer (ICP-MS). With each fluid removal, fresh UHP water was replaced. The calcium concentration was measured using an Agilent Technologies 7900 ICP-MS with an argon plasma. The instrument was configured with a Quartz Micromist nebulizer and Ultra High Matrix Introduction (UMHI) spray chamber. The sample introduction system was set with a 1 L/min flow rate with no dilution. The calibration and analysis were performed by monitoring the calcium 44 isotope.

Mechanical testing. The mechanical properties of the EVA composites were evaluated using a Tinius Olsen 25ST tensile tester with a 25 kN load cell. Experiments were conducted to study the change in the mechanical properties of the EVA composites before and after dissolution. The as-manufactured samples with a length, diameter, and cross-sectional area of 2 mm, 40 mm, and 3.14 mm^2^, respectively, were tested at 25.4 mm/min.

## 3. Results and Discussion

**Figure 2:**
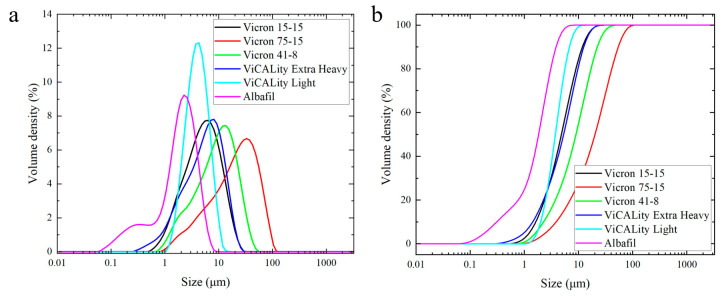
Particle size analysis of CaCO_3_ as-received powders in IPA suspension. Data are presented as volume density as a function of particle size in normalized (**a**) and cumulative normalized (**b**) volume.

**Figure 3:**
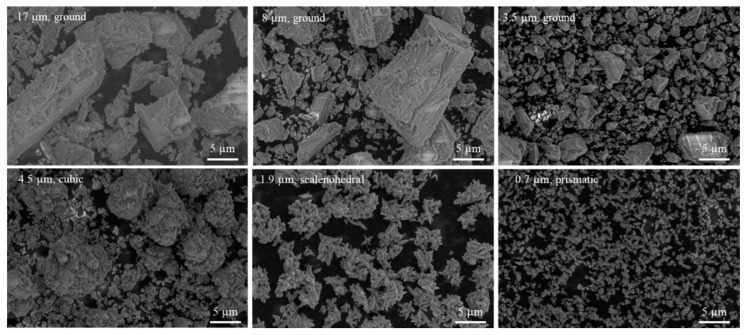
ESEM images of CaCO_3_ as-received powder to confirm particle shape and size prior to compounding.

CaCO_3_ powder was first analyzed for particle size to confirm the size distribution and shape factors to be studied. Figure 2a displays the volume density as a function of particle size for the commercial CaCO_3_ powders. To obtain a better visual representation of cumulative particle size for each material, Figure 2b displays the cumulative volume density for each commercial powder. It is shown that the Albafil (0.7 µm, prismatic) particle has the smallest particle size with the largest size distribution, toting a bi-modal distribution with a large fraction of small particles in the powder. All other samples exhibited a single peak with a long tail. The particle shapes are presented in the SEM micrographs in Figure 3. Because the powder size distribution and geometry has been confirmed, CaCO_3_ and the EVA/CaCO_3_ composites will be identified according to the average particle size/shape, as described in Table 1, for the remainder of this paper.

**Figure 4:**
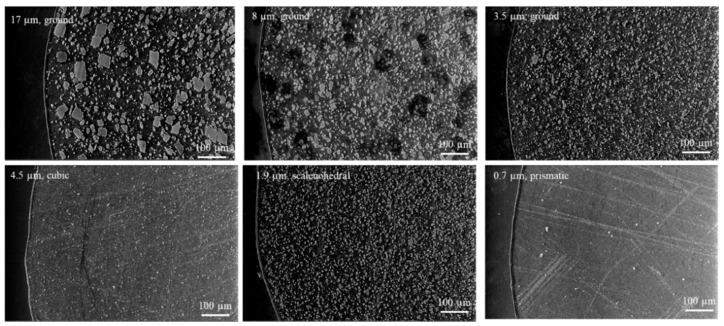
ESEM images of EVA/CaCO_3_ injection-molded implant samples cross-sectioned perpendicular to flow.

The composites were then compounded and pelletized. Samples were fabricated into the implant geometry via injection molding and extrusion. Figure 4 displays the SEM micrographs of the injection-molded implant cross sections for each particle shape/size. It can be seen that there was good dispersion of CaCO_3_ in the polymer matrix, as there was little agglomeration displayed. It is also noted that the CaCO_3_ content from skin to core appeared to be consistent. The 0.7 µm, prismatic CaCO_3_ was so fine and evenly distributed, it was hardly detectable at the scale shown here, and some residual scratching from the polishing protocol was observable. The 8 µm, ground composite appeared to have some pitting at the cross section, where possibly larger CaCO_3_ particles were displaced during the polishing preparation. The injection-molded samples also showed good packing density, as no voids were visible in these cross sections.

**Figure 5:**
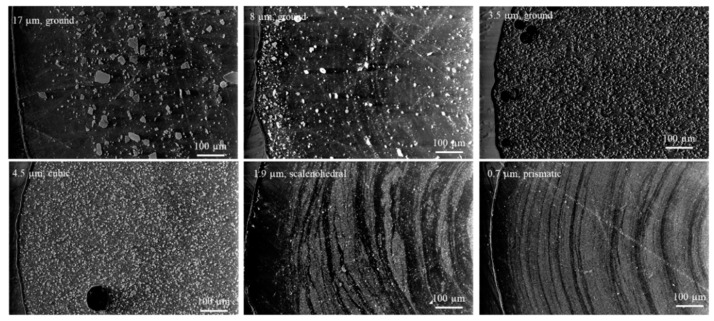
ESEM images of EVA/CaCO_3_ extruded implant samples cross-sectioned perpendicular to flow.

In comparison, the extruded implant geometries are displayed in Figure 5. Similar to the injection-molded samples, there was good dispersion of CaCO_3_, noted by minimal agglomeration. There was also an observable difference in CaCO_3_ content from skin to core across the shear rate gradient of the part, as some samples such as 17 µm, ground composite showed lower CaCO_3_ content at the skin. Flow lines, indicating changes in CaCO_3_ distribution could also be identified in the 0.7 and 1.9 µm CaCO_3_ composites. There were more voids apparent in the extruded specimens, likely due to the lower pressure achievable during the extrusion process. It is also notable that the surfaces of the injection-molded specimens were smoother than that of the extruded specimens, as the injection-molded samples conformed to the surface of the machined core/cavity. The surface imperfections of the extruded samples may be affected by die imperfections, centering tools as the extrudate was drawn through the water bath, or deformations from the weight of the puller.

**Figure 6:**
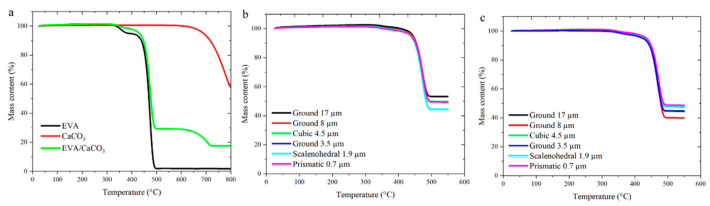
TGA analysis of CaCO_3_ powder and neat EVA resin as a degradation benchmark (**a**), injection-molded composites (**b**), and extruded composites (**c**).

TGA was then used to confirm CaCO_3_ content in the fabricated samples. In this experiment, the mass content at the different degradation temperatures can be used to compare CaCO_3_ and EVA contents. As an example of the different phases, Figure 6a shows the mass content as a function of temperature for the neat EVA, the CaCO_3_ powder, and the EVA/CaCO_3_ composite. It can be seen that the EVA began decomposing at approximately 325 °C and by 500 °C had only about 2% mass left, which can be attributed to leftover char in the crucible. CaCO_3_ had a higher degradation temperature, where thermal decomposition began at approximately 600 °C. To determine the CaCO_3_ contents in the fabricated samples, a heating profile up to 550 °C was then employed, as the polymer degradation fraction could be used to determine CaCO_3_ content. Three samples of each composite were analyzed to obtain an average and a measure of variation, and the average measurements for the injection-molded and extruded samples are shown in Figure 6b,c, respectively. Tabulated CaCO_3_ contents in each formulation are summarized in Table 2. CaCO_3_ contents in the final samples yielded a range from 37.5 to 51.1%, with the goal being 50% by weight. The average CaCO_3_ mass content in the extruded and injection-molded samples were 44.4 and 49.0%, respectively, indicating that pulling the extruded strand through a water bath to cool the profile geometry could result in the loss of some surface CaCO_3_, whereas injection molding allowed the composite to remain fully intact. This was anticipated to have an effect on the burst release when comparing the two manufacturing methods, as the burst release from the extruded implants would not be as strong without the surface CaCO_3_ present.

**Figure 7:**
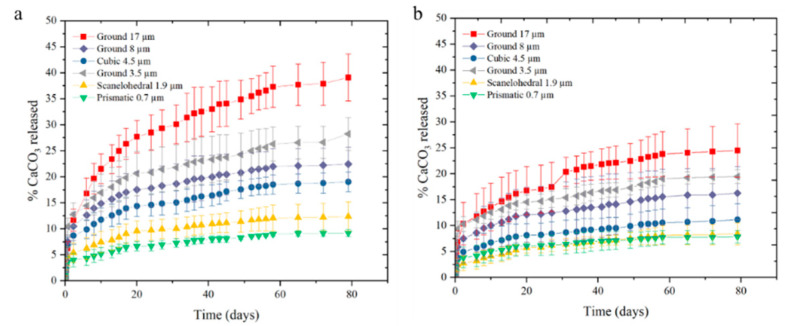
Release of CaCO_3_ from the implant geometry as a function of time for the injection-molded (**a**) and extruded (**b**) samples. The insert shows the burst rate kinetics of CaCO_3_ within the first 2.5 h. Error bars indicate ±1 standard deviation for 3 sample repetitions, and the data point is the average value.

The dissolution of CaCO_3_ from the polymer matrix was then studied over a span of 80 days to measure the content of CaCO_3_ released. The UHP water was measured for Ca content using an ICPMS. A power law model [22] was applied to analyze the content of CaCO_3_ released using the following equation:

(1) $$f_{1}=\frac{M_{i}}{M_{\infty }}=Kt^{n}$$

where *f*_1_ is the amount of drug released, and *M_i_* and *M_∞_* are the amount of drug released over time and the total amount of CaCO_3_ content, respectively. For each composite formulation, *M_∞_* is updated based on the TGA results to reflect the accurate amount of CaCO_3_ in each sample. *K*, *t*, and *n*, represent a constant, time, and an exponent of release, respectively. The dissolution kinetics are displayed in Figure 7 for the injection-molded (a) and extruded (b) samples. It is shown that the burst release, or rapid CaCO_3_ dissolution within the first few days, of the injection-molded samples were greater than that of the extruded counterparts for all formulations studied here. The maximum release was also greater when the sample parts were injection-molded. This is likely due to higher packing pressures achievable during processing, resulting in a denser matrix. Related to the SEM micrographs of the cross sections of injection-molded and extruded rods, the samples manufactured via extrusion contained more voids than injection molding, indicating that the injection molding process could produce a more consistent and denser composite product.

Regarding particle geometry, the release rate proved to have a stronger correlation with particle size than surface area. A larger particle size proved to yield a higher content of CaCO_3_ released, but resulted in a higher variation than the smaller particles. It is assumed that a two-phase mechanism is responsible for this behavior. First, particles on the surface can mechanically separate from the composite rod. This causes a larger burst effect in the samples containing CaCO_3_ of larger particle size. This is also seen as an effect when comparing processing method, as surface CaCO_3_ in the extruded samples is removed during the manufacturing process, as the extrudate is drawn through the water bath. In the second step of the mechanism, CaCO_3_ dissolution is reliant upon the solubility of the compound and the void network creating a pathway to the external solution [6].

DSC was then employed to measure the crystalline content of the EVA phase for each formulation to see if the polymer microstructure would affect dissolution of CaCO_3_. To determine crystallinity, the polymer mass fraction was corrected based on the TGA polymer content summarized in Table 2 to obtain an accurate measure of enthalpy from the polymer fraction. The average absolute crystallinity value for extruded and injection-molded samples, summarized in Table 3, were 34.05 and 14.08%, respectively. The injection-molding process provided a faster cooling rate compared with extrusion, which reduced the time and hindered the kinetics to allow for crystallization. Neat EVA has been shown under similar conditions to crystallize to a higher degree in extrusion compared with injection molding [21]. Within each subgroup, there appeared to be little effect on absolute crystallinity from CaCO_3_ particle size or shape at 50% loading, as no trends were apparent. Prior research agrees that a lower crystalline content in the polymer fraction will increase the release rate [19].

**Figure 8:**
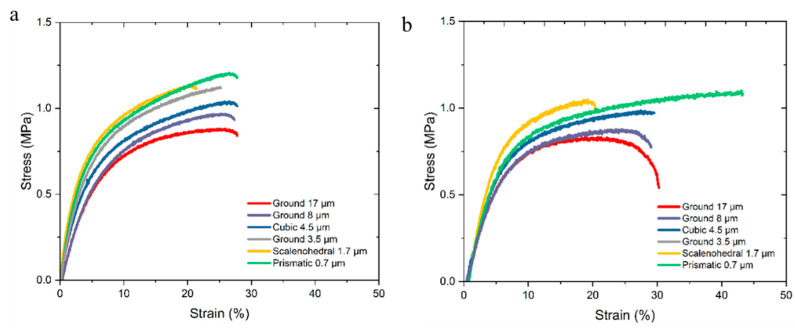
Composite tensile properties of injection-molded (**a**) and extruded (**b**) implant profiles.

Mechanical properties of the material formulations were analyzed by tensile testing a 40 mm-long section of the filament. Average stress/strain behavior for each formulation is displayed in Figure 8. As a general behavior, extrusion proved to permit higher elongation at break than the injection molding fabrication method, as extrusion provided for a higher degree of chain orientation along the main axis. The injection-molded samples also showed a higher average modulus than the extruded samples due to higher achievable packing density from the process. Within each subgroup, a trending behavior showed that a larger particle size resulted in a lower value of maximum stress, due to larger void development as CaCO_3_ particles shifted during plastic deformation.

## 4. Conclusions

The release of CaCO_3_ as a surrogate to an API was tested in experimental pharmaceutical implants. It was found that the manufacturing process used to fabricate the composite samples was a critical parameter for release, as injection molding yielded a higher burst release compared with extruded samples. This is a consequence of the higher packing pressures and resulting densities inherent to the injection molding process. The cooling mode associated with hot melt extrusion includes drawing the filament through a water bath, which was shown to remove the surface CaCO_3_ content, resulting in a lower burst release. When studying the implications of differing particle geometry, CaCO_3_ particle size proved to have a greater impact than the surface area or the particle shape. The release kinetics of this low-solubility compound appeared to be controlled in two phases, where first the surface CaCO_3_ was released by mechanical separation from the EVA by dissolution. Then, the internal network of CaCO_3_ required dissolution to travel through the EVA matrix and through an open void network. The authors hope that this work will help to engineer the next generation of long-acting pharmaceutical-drug-delivery devices, as the release kinetics can be tailored to the desired profile for the application.

## Figures and Tables

**Table 1:** CaCO_3_ grade specifications per Specialty Minerals.

	Vicron 75-17	Vicron 41-8	Vicron 15-15	ViCALity Extra Heavy	ViCALity Light	Albafil
Average Particle Size (µm)	17	8	3.5	4.5	1.9	0.7
Surface Area (m^3^/g)	0.7	1.5	3.2	2.4	7.8	7
Particle Shape	Ground	Ground	Ground	Cubic	Scalenohedral	Prismatic

**Table 2:** TGA experimental findings of CaCO_3_ content.

	CaCO_3_ Content (%)	
CaCO_3_ Geometry	Extruded	Injection Molded
3.5 µm, ground	47	50
17 µm, ground	41	51
8 µm, ground	37	50
4.5 µm, cubic	48	50
1.9 µm, scalenohedral	47	44
0.7 µm, prismatic	46	49

**Table 3:** Crystallinity content as determined by DSC. Values indicate an average of 3 repetitions and ±indicate 1 standard deviation to measure variation.

	% Crystallinity	
CaCO_3_ Geometry	Extruded	Injection Molded
17 µm, ground	33.92 ± 1.59	13.71 ± 1.92
8 µm, ground	40.43 ± 1.98	15.84 ± 0.42
4.5 µm, cubic	30.23 ± 0.21	13.34 ± 1.45
3.5 µm, ground	33.85 ± 2.60	13.24 ± 2.74
1.9 µm, scalenohedral	33.43 ± 3.50	14.26 ± 5.55
0.7 µm, prismatic	32.45 ± 1.80	14.06 ± 1.95

## Data Availability

The data that support the findings of this study are available from the corresponding author, upon reasonable request.
